# Hyponatremia and V2 vasopressin receptor upregulation: a result of HSP90 inhibition

**DOI:** 10.1007/s00280-017-3390-x

**Published:** 2017-08-04

**Authors:** Qiong Yang, Florian Puhm, Michael Freissmuth, Christian Nanoff

**Affiliations:** 1 Centre for Physiology and Pharmacology, Institute of Pharmacology, Medizinische Universität Wien, Währingerstraße 13A, 1090 Vienna, Austria; 2 Gaston Glock Laboratories for Exploratory Drug Research, Centre for Physiology and Pharmacology, Medizinische Universität Wien, Währingerstraße 13A, 1090 Vienna, Austria

**Keywords:** Hyponatremia, HSP90 inhibitor, V2 vasopressin receptor, Upregulation, HELA cells

## Abstract

**Purpose:**

Small-molecule inhibitors of heat-shock protein 90 (HSP90) have been under development as chemotherapeutic agents. The adverse events reported from early clinical trials included hyponatremia. Given the limited number of patients enrolled, the number of hyponatremia incidents was remarkable and repeatedly, the event was judged as severe. Inappropriate V2 vasopressin receptor stimulation is an established cause of hyponatremia. We explored the hypothesis that HSP90 inhibition produces hypersensitivity to vasopressin by upregulating V2-receptors.

**Methods:**

Experiments were carried out in cell culture using HEK293 cells with heterologous expression of the human V2-receptor and HELA cells with an endogenous V2-receptor complement. We tested the effect of HSP90 inhibition by three structurally unrelated compounds (alvespimycin, luminespib, radicicol) and asserted its specificity in cells depleted of cytosolic HSP90 (by RNA interference). Assays encompassed surface V2-receptor density and vasopressin-stimulated formation of cyclic AMP (cAMP).

**Results:**

The results demonstrate a twofold increase in cell-surface receptor density following pre-incubation with each of the HSP90 inhibitors. The effect had a concentration-dependence consistent with the individual potencies to inhibit HSP90. Similarly, depletion of cytosolic HSP90 increased surface-receptor density and at the same time, reduced the inhibitor effect. Upregulated V2-receptors were fully functional; hence, in culture treated with an HSP90 inhibitor, addition of vasopressin resulted in higher levels of cAMP than in controls.

**Conclusion:**

Since formation of cAMP is the first signalling step in raising water permeability of the collecting duct epithelia, we suggest that V2-receptor upregulation generates hypersensitivity to vasopressin linking HSP90 inhibition to the development of hyponatremia.

## Introduction

The original finding that preceded the introduction of HSP90 inhibitors as chemotherapeutic agents was the observation that benzoquinone ansamycins restored the phenotype of cells transformed with oncogenic tyrosine kinases [1]. The predominant cellular binding site of the benzoquinone ansamycins such as geldanamycin is the ATPase domain of HSP90, the occupancy of which impairs the activity of protein kinases, a major class of binding partners of cytosolic HSP90 (reviewed in [2]).

Although targeting HSP90 has shown promise in preclinical tumour models, the inhibitors unfortunately were of limited efficacy in the clinic, presumably because of rapidly emerging resistance. A strategy has been to overcome resistance by introducing chemically unrelated inhibitory compounds. From recent phase I/II clinical trials that enrolled patients with various kinds of cancer, a good number reported on hyponatremia as adverse event; these reports are summarized in Table 1. With about 380 patients treated over short time, the event count was 60. The severity grades suggest that in some instances hyponatremia may have been dose-limiting. Given the structural diversity of the compounds employed (half of which were not benzoquinone ansamycins) an off-target effect was unlikely relevant. Therefore, our conjecture was that HSP90 inhibition is causally linked to the aetiology of hyponatremia.

**Table 1:** Hyponatremia incidence in phase I/II trials of HSP90 inhibitors

HSP90 inhibitor	Number of events	Number of patients enrolled	Severity grade	References
17-DMAG	1	11	1	Pacey et al. [3]
17-AAG	4	25	1, 3	Pacey et al. [4]
17-AAG (plus docetaxel)	27	49	1, 3	Iyer et al. [5]
17-AAG (plus bortezomib)	1	11	≥3	Walker et al. [6]
IPI-504	2	19	≥3	Oh et al. [7]
SNX-5422	1	33	1	Rajan et al. [8]
Ganetespib	3	53	≥3	Goldman et al. [9]
Debio0932	2	80	≥3	Isambert et al. [10]
HSP990	2	64	≥3	Spreafico et al. [11]
Luminespib (plus erlotinib)	17	37	1, 3	Johnson et al. [12]
Onalespib	2	31	1, 3	Do et al. [13]
Ganetespib	1	17	3	Thakur et al. [14]

While it is largely unknown how HSP90 selects its clients, due to its physical abundance it is certainly poised for engaging cellular proteins as binding partners. The cytosolic isoforms (HSP90α/HSP90AA1 and HSP90β/HSP90AA2) make up for a significant fraction (>1%) of the mass of cytoplasmic proteins. It is therefore not surprising that experimental evidence has revealed an increasing number of cellular functions HSP90 impinges on. A few studies have addressed the effect of HSP90 on signalling-competent G-protein-coupled receptors and reported an increase with a decline in HSP90 activity [15, 16].

The V2 vasopressin receptor is a Gs-coupled receptor with a singular action in kidney physiology. Activation by its agonist restrains the excretion of water. Excessive V2-receptor activation is the cause of hyponatremia as in the case of inappropriate vasopressin secretion (SIADH) and may herald water intoxication. Therefore, we tested the assumption that treatment with HSP90 inhibitors sensitizes cells to the V2-receptor-mediated effect of vasopressin. In addition to the prototypical HSP90-inhibitor DMAG (alvespimycin), we used two chemically unrelated compounds (radicicol and luminespib). DMAG and luminespib had been used in clinical trials where hyponatremia occurred as an adverse event (Table 1). Our experimental evidence demonstrates that HSP90 inhibition increased V2-receptor levels and enhanced vasopressin signalling consistent with the hypothesis that HSP90 inhibition predisposes to hyponatremia by upregulating the V2-receptor.

## Materials and methods

A polyclonal antibody that recognizes both HSP90α and β isoforms (HSP90AA1 and AB1) and a monoclonal directed against GAPDH (glyceraldehyde 3-phosphate dehydrogenase) were from Cell Signaling Technology. The Alexa Fluor 488-conjugated F(ab’)2 fragment of goat anti-mouse IgG was from LifeTechnology,

The radiotracers [^3^H]adenine and [^3^H]arginine-vasopressin were purchased from PerkinElmer (Waltham, Massachusetts). SR121463 (SR) was kindly made available by Dr. Claudine Serradeil (Sanofi-Aventis, Toulouse, France). Luminespib (NVP-AUY922) was from Selleckchem (Munich, Germany).

The following reagents were from Sigma-Aldrich: M2-monoclonal anti-FLAG antibody, analytical grade 17-dimethylaminoethylamino-17-demethoxygeldanamycin (DMAG), radicicol, forskolin, arginine–vasopressin (AVP) and primer oligonucleotides. Restriction enzymes were from Roche-Diagnostics (Mannheim, Germany), Pfu-DNA-Polymerase from Agilent (Santa Clara, California), chromatography resin for DNA purification from Macherey-Nagel (Düren, Germany). All other reagents were of the highest quality grade available.

Stock solutions of SR121463, DMAG and radicicol were in dimethyl sulfoxide (DMSO) at concentrations of 10 mM (luminespib 2 mM). We have ruled out vehicle effects by testing DMSO up to a maximum concentration of 0.1% (not shown).

The human V2-receptor cDNA subcloned in the peGFP-vector to give a c-terminally appended GFP (green fluorescent protein) was a gift of Dr. Ralf Schülein (Leibniz Institute of Molecular Pharmacology, Berlin, Germany). By inserting the receptor cDNA into the pCMV-Tag2B vector (Stratagene, La Jolla, California) a FLAG-epitope sequence was appended to the 5′-end of the V2-receptor coding sequence. The integrity of the construct was verified by fluorescent sequencing.

### Cell culture and transfection

We used receptor-transfected HEK293 cells propagated in the presence of G418 and stocked in aliquots. Results obtained with this type of culture were indistinguishable from experiments performed after transient transfection (not shown).

HEK293 cells and JEG-3 cells were cultivated in DMEM plus l-glutamine and 10% foetal calf serum. The transfection reagent was JetPEI (Polyplus, Illkirch, France) and was used for transfection with plasmid cDNA and with siRNA oligonucleotides. Culture medium for HELA-S3 cells (HELA) was RPMI 1640 containing foetal calf serum and l-glutamine. JEG-3 cells were used after transient transfection with the receptor expression plasmid.

HELA cells were from the Institute of Cancer Research (Comprehensive Cancer Center Vienna, Medical University of Vienna), JEG-3 cells were a kind gift of Dr. Martin Knöfler (Department of Obstetrics and Gynaecology, Reproductive Biology Unit, Medical University of Vienna).

Ambion small interfering RNA (siRNAs) directed to human HSP90α (HSP90AA1) and HSP90β (HSP90AB1), consisting of two species of oligonucleotides each, and scrambled-sequence control oligonucleotides were from LifeTechnologies. Routine siRNAs concentrations during transfection were 15 nM. HEK293 cells transfected with siRNAs were subjected to assay 4 days later.

### Determination of surface-receptor density

HEK293 cells expressing a V2-receptor construct with an N-terminal FLAG-epitope were incubated with HSP90 inhibitors routinely overnight, compounds added to growth medium. After washing with phosphate buffered saline (PBS) cells were detached in EDTA (0.02%) solution. All of the following procedures were performed at 4 °C. Cells were collected by centrifugation and taken up in PBS with 1% BSA in a volume of ~0.3 mL. Cell surface-receptors were labelled with the monoclonal M2 anti-FLAG antibody (at a concentration of ~0.3 µg/100 µL cell suspension) and a secondary anti-mouse IgG conjugated to fluorescent dye (AlexaFluor 488, 0.25 µg/100 µL). Labelling was for a total of 40 min with the primary and for the last 20 min included the secondary antibody. Cells were washed once, resuspended in PBS with 1% BSA and were expedited to measuring cell-bound AlexaFluor 488 fluorescence by flow cytometry.

For flow cytometry, we used a Becton-Dickinson instrument (FacsCanto). Recordings were at 4 °C. For analysis, a cell fraction was gated with forward scatter values higher than 10% of maximum. This fraction was set off from a fraction of non-viable cells stained positive for propidium iodide (not shown). Recording comprised ten thousand events per gate. Receptor-specific fluorescence was determined after excluding events that overlapped the intensity range of non-specific fluorescence (the lower 97% of events recorded on non-transfected HEK293 cells labelled with primary and secondary antibody). The number of events representing the number of cells with the FLAG-tagged receptors on their surface was between 4000 and 8000. Original data are plotted in histograms with the fluorescence intensity on the *x*-axis and the event count on the *y*-axis. Overlay histograms were normalized to the peak event count (given as *y*-axis label).

We quantified treatment-dependent changes by comparing the area under the histogram trace marking the distribution of receptor-specific fluorescence events or used the median value of receptor-specific fluorescence intensity for gauging treatment effects. Analysis of flow cytometry recordings was performed with the use of Flowing Software (Dr. Perttu Terho, Turku Centre for Biotechnology, Finland).

### Visualization of cells expressing GFP-tagged V2-receptor

HEK293 cells expressing the wild-type receptor fused to GFP were seeded on poly-d-lysine-coated glass bottom dishes. Four days after transfection with siRNA, GFP-fluorescence was recorded with a confocal laser-scanning microscope (Zeiss LSM510). Pinhole was set to limit slice thickness to less than 2.5 µm.

### Determination of cellular cAMP formation

Formation of cAMP was determined in cells pre-incubated with [^3^H]adenine. The assay incubations were in Krebs–Henseleit buffer plus an inhibitor of phosphodiesterase (0.1 mM Ro 20-1724) at room temperature, started with the addition of forskolin, the receptor agonist or both as indicated (incubation time was 15 min). Tritiated cAMP was separated chromatographically from radioactive nucleotides and adenine according to [17]. When assays followed a pre-incubation of cells with an HSP90 inhibitor, we allowed the compound to dissociate for a period of 40 min and washed it out before the assay.

### Miscellaneous procedures

Binding of [^3^H]AVP was assayed on a membrane fraction prepared from HEK293 cells transfected with the V2-receptor expression plasmid. Incubation proceeded at room temperature for 90 min in the presence of 50 µg membrane protein according to [18]. Separation of bound and free radioligand was by filtration over glass fibre filters soaked in 0.3% polyethyleneimine. The amount of radioligand bound in the presence of 1 µM SR121463 defined the fraction of non-specific binding; this was ~20% of total binding in the KD concentration range.

Protein concentration in cell fractions was determined with the Bio-Rad Bradford dye.

Statistical analysis was done by repeated-measure anova, which compared treatment-dependent changes within single experiments, or by Kruskal–Wallis anova followed by a post hoc test to compare group means. Where appropriate, we compared means of experimental values between two groups using *t* test or Mann–Whitney rank-sum tests. Data obtained with graded concentrations of HSP90 inhibitors or AVP were subjected to non-linear curve fitting according to the following equation, *f* = *y*0 + a*x/(*b* + *x*), where *y*0 is *y* at concentration *x* = 0; *a* is the maximum effect; *b* is the EC50; and *x* is the inhibitor concentration. The curve generated by the fitting procedure represents a rectangular hyperbola.

## Results

**Fig. 1:**
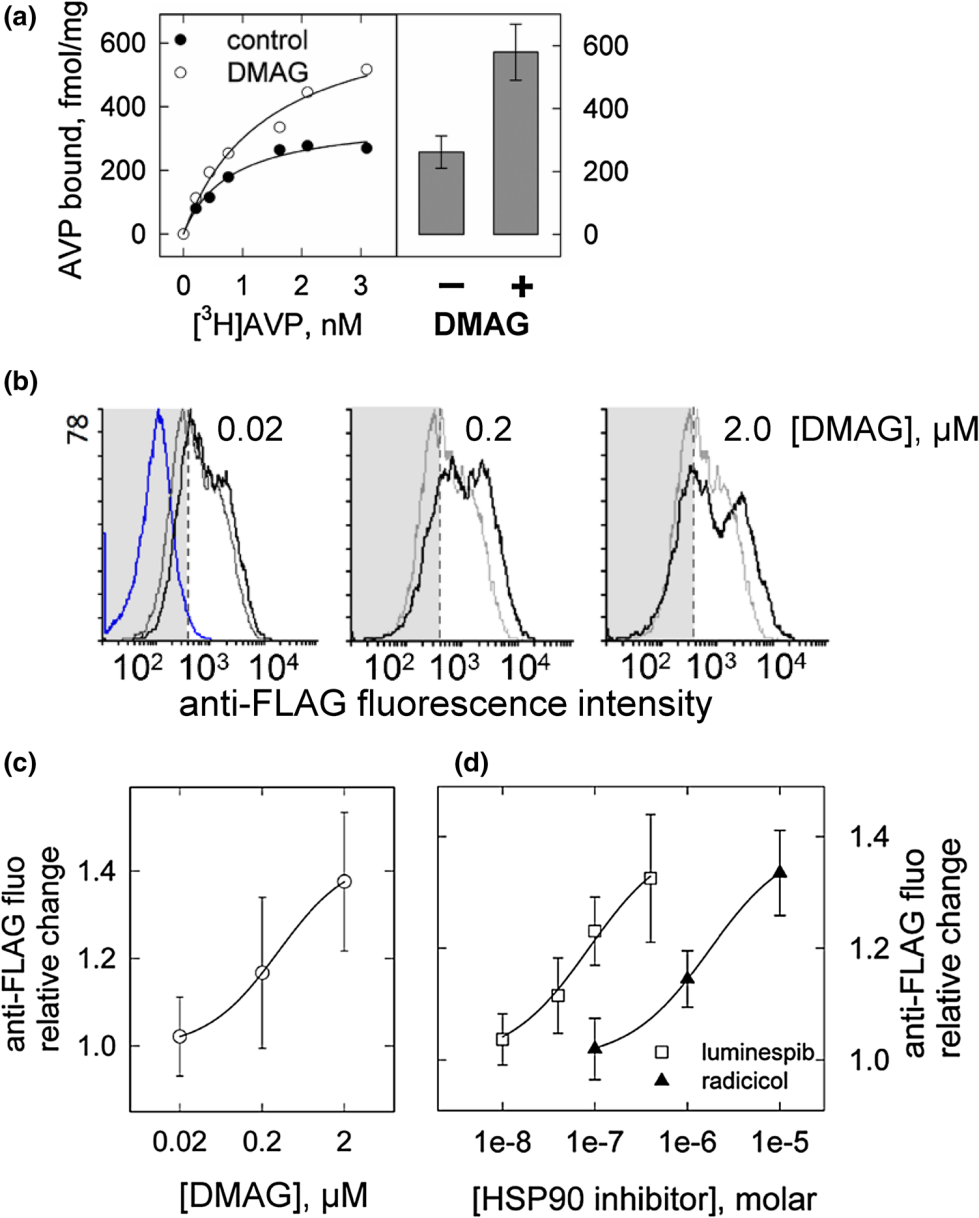
Inhibition of HSP90 increased V2-receptor density at the cell surface. **a** Representative [^3^H]AVP binding isotherms (specific binding) from an experiment carried out on membranes from cells transfected to express the V2-receptor and pre-incubated with DMAG (2 µM, *open circle*) or vehicle (*filled circle*). The *bar graph* depicts means of Bmax values estimated by curve fitting of the data from three independent [^3^H]AVP binding experiments. *T* test confirmed a significant difference between membranes from DMAG-treated and untreated culture. **b** Concentration-dependent effect of DMAG on V2-receptor surface density. Shown are histograms representing original recordings by flow cytometry of anti-FLAG antibody-labelled cells. *Black traces* represent a 20-h incubation with DMAG at the indicated concentrations, *grey traces* untreated cells. The *grey-shaded* area delimits the distribution of the fluorescence signal obtained from non-transfected cells labelled with antibody; the left-hand panel includes the respective histogram (*blue trace*) for representation purpose. **c**, **d** Quantification of surface receptor-specific fluorescence. The effect of the HSP90 inhibitors DMAG (open circle, **c**), luminespib (*open square*) and radicicol (*filled triangle*) (**d**) is given as change of the median of fluorescence intensity relative to vehicle-treated controls (*n* = 4–8, *error bars* = s.d.). For each HSP90 inhibitor, the increase due to the highest concentration was significantly different from the respective control values (*p* < 0.05, Kruskal–Wallis ANOVA and Dunn’s test)

We assessed cell surface expression of the human V2 vasopressin receptor after pre-treatment with the benzoquinone ansamycin DMAG and of two structurally unrelated inhibitors of HSP90, respectively. The number of V2-receptors we determined by radioligand binding and by antibody-labelling of the FLAG-epitope attached to the receptor N-terminus. Figure 1 demonstrates that HSP90 inhibition increased the cell surface density of the V2-receptor. Figure 1a depicts specific binding of [^3^H]AVP to membranes prepared from HEK293 cells transfected with an expression vector coding for the human V2-receptor. Cell culture was incubated for 20 h with 2 µM DMAG (open symbols in Fig. 1a) or vehicle (0.1% DMSO, closed symbols). In membranes from DMAG-treated cells, the amount of [^3^H]AVP bound was higher than in membranes from controls; fitting of the data indicated that DMAG incubation increased Bmax estimates by about twofold with no change in affinity for [^3^H]AVP (controls KD = 1.08 ± 0.65 nM, DMAG-treated = 1.26 ± 0.29 nM, means ± s.d., *n* = 3).

The relative increase was in the same range when FLAG-tagged receptors were labelled with antibody recognizing the cognate epitope. Antibody-labelling was done on ice, which precluded uptake of primary and secondary antibody. Figure 1b shows original histograms from a set of individual recordings by flow cytometry. The grey trace represents vehicle-treated cells, the black trace cells after pre-incubation with DMAG at the concentrations indicated (0.02, 0.2, 2 µM). The grey-shaded area delimits the fluorescence signal of antibody-labelled untransfected cells (the non-specific signal) and covers ~97% of the events recorded (indicated by the blue trace). It is evident that DMAG shifted the black trace to higher fluorescence values. The area under the trace—reflecting the number of labelled receptors—increased by approximately twofold at the highest DMAG concentration. At higher concentrations of DMAG, there was an additional effect due to enhanced non-specific fluorescence (Fig. 1b). We therefore refrained from escalation of DMAG-concentrations beyond 2 µM and used the median of the fluorescence intensity distribution as a conservative estimate (rather than the area under the trace) for the quantification of differences. Changes in the median of fluorescence intensity are plotted in Fig. 1c. They show that DMAG was effective at submicromolar concentrations after an overnight incubation (EC50 = 0.33 ± 0.24; estimate ± standard error), which is consistent with its affinity for HSP90 [19]. An extended incubation period (16–20 h) was necessary while 2 h produced no effect (data not shown).

In addition to DMAG, we tested radicicol, a cyclic polyketide and luminespib, a non-cyclic resorcinylic isoxazole amide. Antibody-labelling of the V2-receptor revealed a concentration-dependent increase of surface-receptor density by radicicol and luminespib. A plot of the median values of bound antibody fluorescence is shown in Fig. 1d. Fitting of the data to a rectangular hyperbola gave individual EC50 estimates of 0.08 ± 0.03 and 1.7 ± 0.7 µM for luminespib and radicicol, respectively; the maximum increase was similar to that produced by the highest concentration of DMAG.

### Depletion of cytosolic HSP90 increases surface-receptor density

**Fig. 2:**
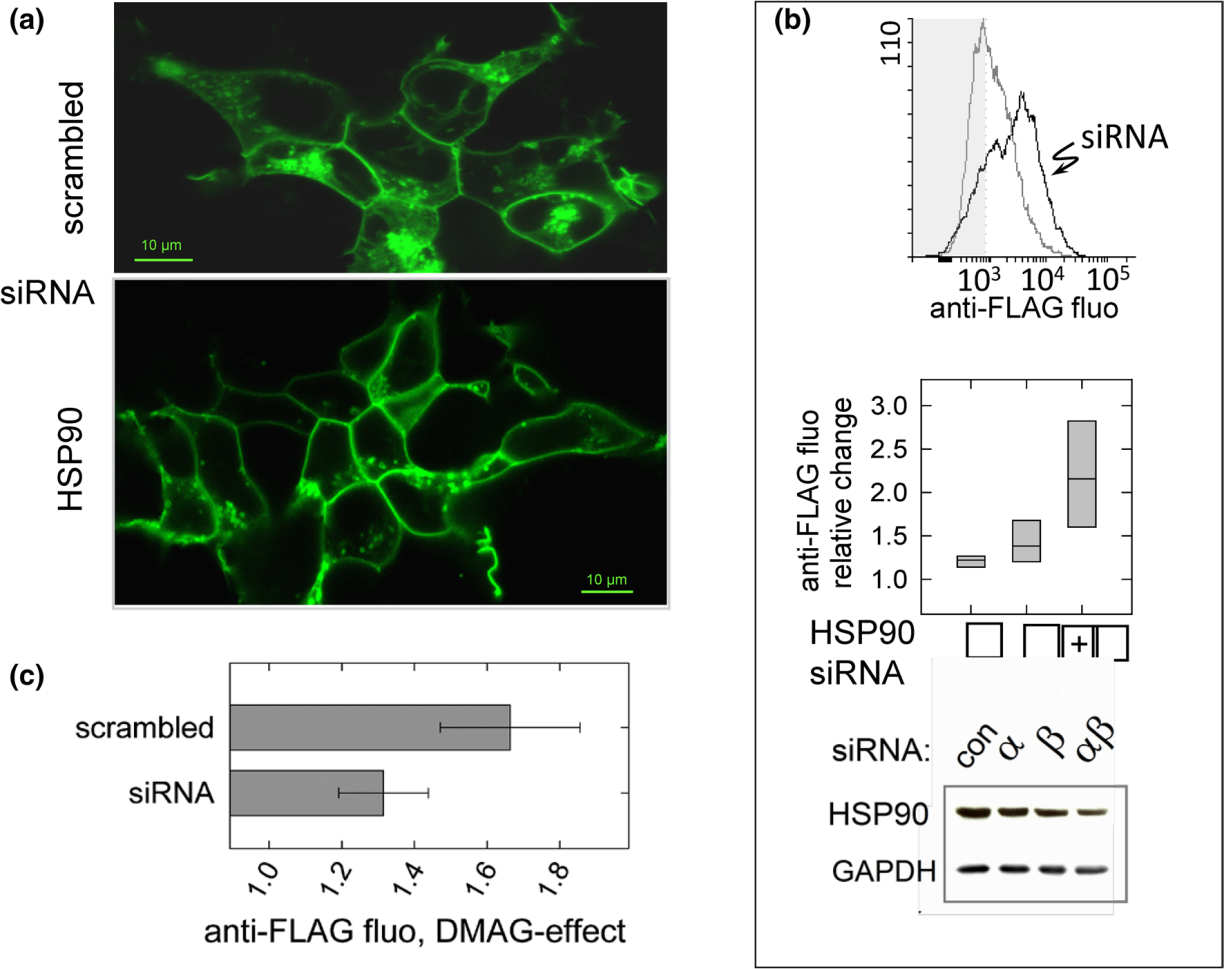
Effect of siRNAs directed against cytosolic HSP90 isoforms on the V2-receptor cell surface density. **a** HEK293 cells expressing the GFP-tagged V2-receptor were transfected with siRNAs directed against HSP90 α-and β-isoforms or with scrambled-sequence control oligonucleotides as indicated. The confocal images (optical slice thickness <2.5 µm) depict the subcellular distribution of the fluorescent receptor. **b** Histogram from an original recording of cells transfected with siRNAs against HSP90 α/β (*black trace*) or with control oligonucleotides (*grey trace*) followed by antibody-labelling of the FLAG-tagged V2-receptor. Box plots document the change of receptor fluorescence from three experiments with siRNAs against HSP90α, HSP90β or both (*horizontal line* = median; *borders* = 25 and 75% confidence intervals). Statistical comparison was by repeated measures ANOVA; the effect of combined siRNAs was statistically significant versus control (Holm–Sidak test). Western blots show levels of HSP90 in a cytosolic fraction determined after transfection with control (scrambled-sequence oligonucleotides), isoform-specific or with a 1-to-1 combination of α- and β-specific siRNAs. The antibody used to detect HSP90 was directed against both α- and β-isoforms. The blot was probed for GAPDH as loading control. The Western blot shown is representative of two transfection experiments. **c** DMAG-effect on the V2-receptor after transfection with siRNAs. Effect on receptor surface density was measured by antibody-labelling and flow cytometry; *horizontal bars* represent means (±s.d.) of the relative change in the medians of fluorescence intensity, which were significantly different between scrambled and HSP90-specific siRNAs (paired *t* test)

RNA interference (RNAi) confirmed regulation of cell surface receptor density by cytosolic HSP90. We employed four different siRNA oligonucleotides, two directed against each isoform of cytosolic HSP90, HSP90α and HSP90β. Four days after transfecting HEK293 cells with the siRNAs, we recorded confocal images of the GFP-tagged V2-receptor (Fig. 2a) and measured receptor density after antibody-labelling of the FLAG-tagged construct (Fig. 2b). The micrographs shown in Fig. 2a document a change in the subcellular distribution of the GFP-tagged receptor. The fluorescent lines marking cell contours appeared more pronounced in culture transfected with the HSP90-specific siRNAs (HSP90, bottom micrograph in Fig. 2a) than with scrambled-sequence control oligonucleotides (scrambled, top micrograph in Fig. 2a), while the fluorescent signal within the cells was reduced. This impression was substantiated by quantification of the fluorescence distribution (not shown). Antibody-labelling of the receptor showed that transfection with HSP90-specific siRNAs augmented V2-receptor density at the cell surface (Fig. 2b, histogram), similar to the effect of HSP90 inhibition. The box plots summarize three transfection experiments. If cells were transfected with siRNAs targeting both cytosolic isoforms, HSP90α and β, receptor upregulation was twofold on average and significantly different from control cells transfected with scrambled-sequence siRNAs. Transfection with single-isoform-specific siRNA oligonucleotides was less effective than the combination (Fig. 2b, box plots): they produced small increments where the difference with controls was not significant. We ruled out that the effect of the combined siRNA oligonucleotides was due to higher concentrations of siRNA (15 vs. 30 nM), employed with the combination (data not shown). The bottom panel of Fig. 2b represents a Western blot of cytosolic HSP90 to control for RNAi efficiency. Cytosolic fractions from cells transfected with siRNAs against HSP90 isoforms, α, β or both, were blotted as indicated. It is evident that RNAi reduced the HSP90 band visualized with antibody, which recognized both the α- and β-isoform of cytosolic HSP90; GAPDH was stained for to control for equal loading of the extracts. The decrease caused by transfection with the combination of siRNAs (directed against both isoforms of HSP90) exceeded that seen with single siRNAs against the individual isoforms, similarly to the effect on V2-receptor surface density (Fig. 2b, box plots).

Thus, depletion of cytosolic HSP90 mimicked the effect of chemical HSP90 inhibition. With a reduced level of the target, the inhibitor effect is predicted to diminish. In fact, depletion of HSP90 limited the effect of DMAG: receptor upregulation was less relative to controls, which were cells transfected with scrambled-sequence siRNAs. The bars in Fig. 2c document a significant difference of the increments. This finding further substantiates the assumption that receptor upregulation was due to a decline in the activity of cytosolic HSP90.

### Enhanced V2-receptor signalling following HSP90 inhibition

**Fig. 3:**
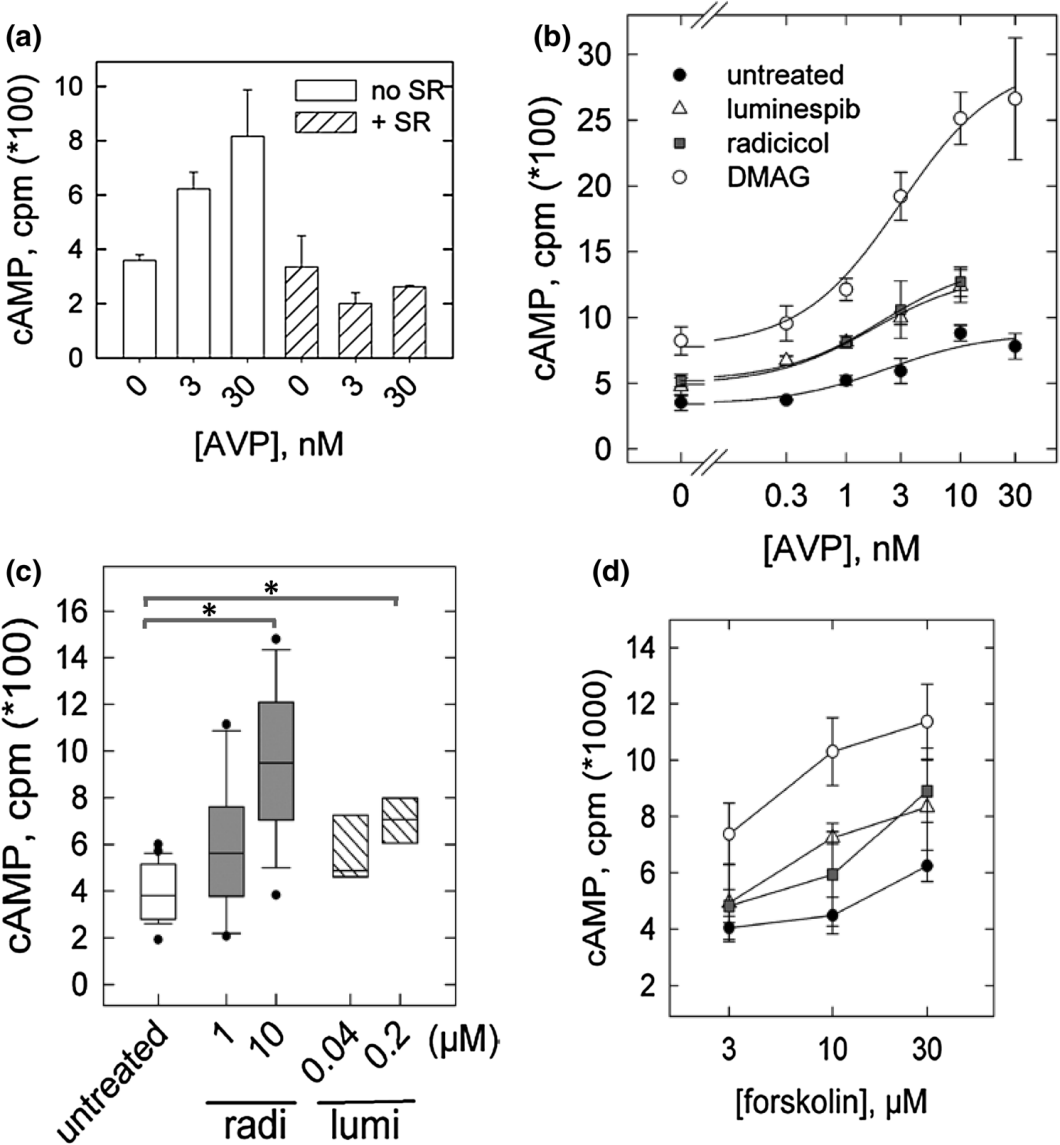
V2-receptor signalling in HELA cells after pre-incubation with HSP90 inhibitors. **a** Formation of cAMP stimulated by AVP (at 3 and 30 nM) in non-transfected HELA cells, suppressed by the addition of the selective V2-antagonist SR121463 (100 nM). To augment adenylyl cyclase sensitivity to stimulation by the Gs-coupled V2-receptor, the AVP-response in HELA cells was determined in the presence of 10 µM forskolin (in this experiment and in the experiments shown in **b**, **c**). **b** Concentration-dependent AVP-response in vehicle-treated HELA cells (*filled circle*) and in cells pre-treated with DMAG (2 µM, *open circle*), luminespib (0.2 µM, *open triangle*) and radicicol (10 µM, *filled square*), respectively. Cells grown to near confluence were labelled with [^3^H]adenine and treated with HSP90 inhibitor overnight. Shown are mean values of cAMP (±s.e.m.) from three to four experiments, each performed in triplicates. Fitting the values to a rectangular hyperbola returned similar EC50-estimates (2–3 nM AVP) after each inhibitor and produced the inserted spline curves. **c** *Box plots* of cAMP values (*n* = four experiments) obtained with a fixed concentration of AVP, which was 3 nM (after a pre-incubation with radicicol) or 1 nM (luminespib). Values were from HELA cells pre-incubated with radicicol and luminespib at graded concentrations (nil, submaximal and maximal). The averaged values from vehicle-treated cells and from cells treated with the maximal concentration (10 µM radicicol, 0.2 µM luminespib) included values shown in **b**. *Asterisks* indicate a significant difference relative to controls (Kruskal–Wallis anova followed by Dunn’s test, *p* < 0.05). **d** Formation of cAMP at various concentrations of forskolin in HELA cells pre-incubated with vehicle (*filled circle*), with DMAG (2 µM, *open circle*), luminespib (0.2 µM, *open triangle*) or radicicol (10 µM, *filled square*). Data represent means from three experiments performed in duplicates (±s.e.m.)

Receptor upregulation translated into enhanced stimulation of cAMP formation by AVP. This was observed in two cell lines, transfected HEK293 and HELA cells, which express the V2-receptor endogenously. Figure 3a shows that HELA cells responded to nanomolar AVP with an increased formation of cAMP. The effect of AVP was completely suppressed by the addition of the V2-selective antagonist SR121436 (at 100 nM). Pre-incubation with the HSP90-inhibitors DMAG, radicicol and luminespib augmented the V2-receptor response. Figure 3b presents the results of a concentration-dependent stimulation by AVP. It is obvious that HSP90 inhibition enhanced cAMP formation shifting the concentration response curves upward. The effect was most pronounced with DMAG with a nearly twofold sensitization of the response; the AVP-dependent increment over basal was 1.7-fold (±0.4) in controls and threefold (±0.6; s.d. from three experiments) in DMAG-treated culture with no apparent change in agonist potency. Pre-incubation with radicicol (10 µM) and luminespib (0.2 µM) resulted in a somewhat smaller enhancement. We repeated the assays with a stepwise increase of the inhibitor concentration to ascertain that the effect by luminespib and radicicol was compatible with HSP90 inhibition. Figure 3c shows box plots representing cAMP formation at a fixed concentration of AVP. The results demonstrate that for both, radicicol and luminespib, the concentrations were similar to those required for increasing the receptor surface density (cf. Fig. 1d). For the maximal concentrations of each, radicicol and luminespib the pooled data indicate a statistically significant increment relative to the controls, which was comparable in size with the DMAG-effect (~twofold).

Figure 3b suggests that HSP90 inhibition also had an effect on the basal rate of cAMP formation (at zero AVP, basal cAMP formation was marginally higher in treated than in untreated culture). Figure 3d shows forskolin-dependent cAMP formation in HELA cells where values were higher in cells pre-incubated with an HSP90 inhibitor. Apparently, the trend was more pronounced after pre-incubation with DMAG (open circle) than with luminespib (open triangle) and radicicol (open square).

**Fig. 4:**
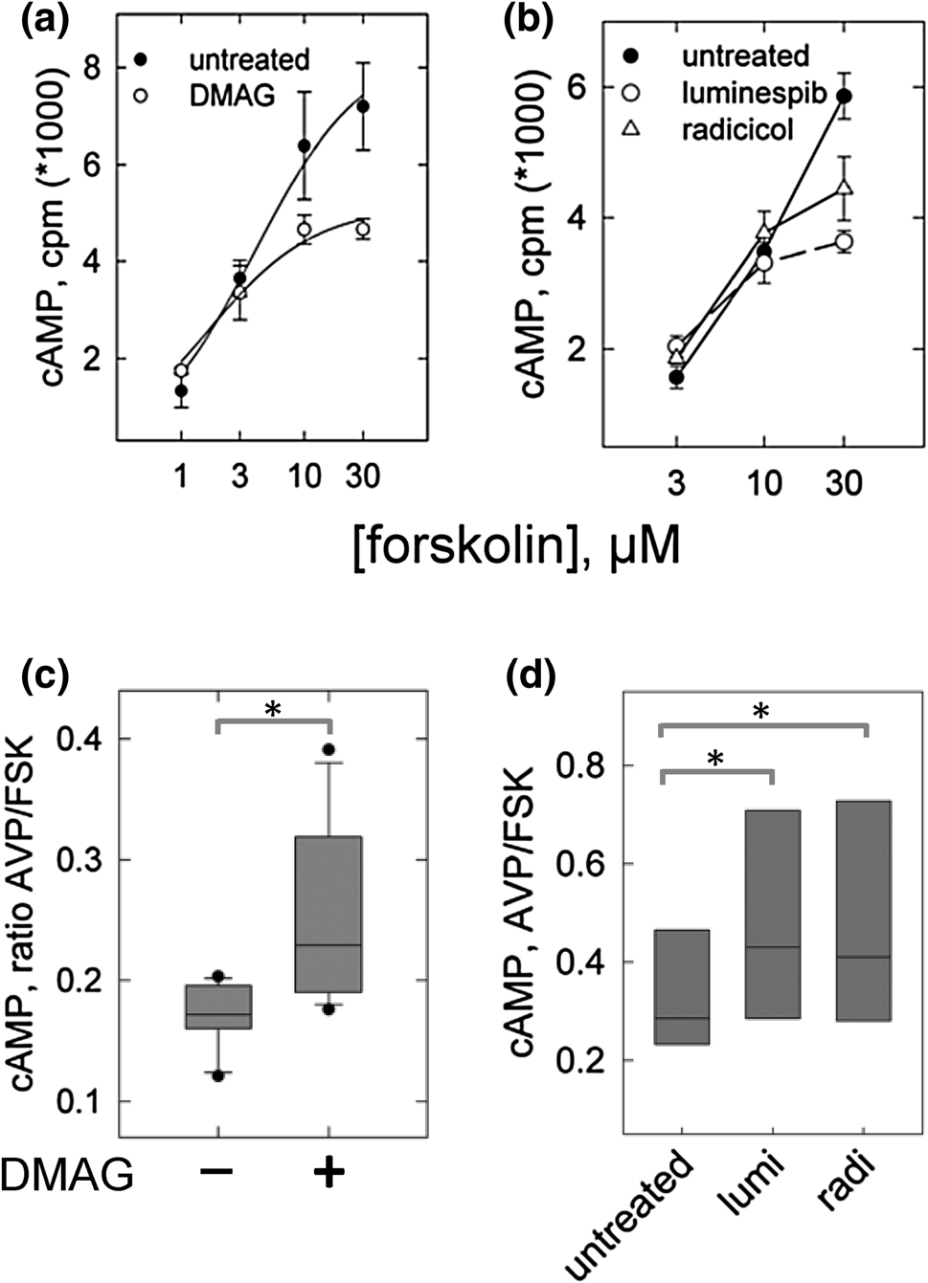
Signalling by the V2-receptor heterologously expressed in HEK293-cells after pre-incubation with HSP90 inhibitors. **a**, **b** Formation of cAMP at various concentrations of forskolin in transfected HEK293 cells pre-incubated in **a** with vehicle (*filled circle*) or DMAG (2 µM, o) and in **d** with vehicle (*filled circle*), luminespib (0.2 µM, *open triangle*) or radicicol (10 µM, *filled square*). Data represent means from two experiments performed in triplicates (±s.e.m.). **c** HEK293 cells transfected with the V2-receptor and treated overnight with DMAG or vehicle were assayed for cAMP on stimulation with AVP (10 nM) and separately, with forskolin (10 µM). The cAMP values obtained with AVP were normalized to those obtained with forskolin (values amounting to between 3000 and 5000 cpm/well) in the same experiment. *Box plots* depict the average of three experiments in quadruplicate determinations (borders representing the 75th and 25th percentiles, whiskers the 90th and 10th percentiles). The difference between control and DMAG-pre-treated cells was confirmed by Mann–Whitney statistics (*p* < 0.05). **d** Cells treated with radicicol (10 µM), luminespib (0.2 µM) or vehicle (untreated) were assayed for cAMP formation. Similar to **c** AVP-mediated cAMP formation is given relative to the cAMP formed in the presence of forskolin in the same experiment (*n* = 4). Repeated measures anova indicated a significant difference (*p* < 0.05)

Increased basal activity of the catalyst was a cell line-specific effect of HSP90 inhibition, not a generic one. In HEK293 culture, for example, pre-incubation of with HSP90 inhibitors slightly reduced catalyst activity. Figure 4a, b shows that in the presence of forskolin at higher concentrations, cAMP formation was somewhat lower than in the controls. An appreciable reason is reduced viability of the HEK293 culture on HSP90 inhibition (HELA cells in contrast appeared resistant to a detrimental effect).

Because we could not be certain of the fraction of cell culture contributing to cAMP formation, we normalized AVP-stimulated Camp formation to basal values; to elevate basal values, we assayed cAMP formation also in the presence of forskolin only (10 µM). Figure 4c documents AVP-stimulated cAMP production in transfected HEK293 cells after DMAG treatment; that is the ratio of AVP values over forskolin values, relative to vehicle-treated cells. The box plots in Fig. 4d show the ratios after pre-incubation with radicicol and luminespib. For each inhibitor the increase was statistically significant and consistent with the greater number of surface V2-receptors after inhibition of HSP90.

## Discussion

Preclinical drug profiling allows for predicting a range of side effects, but there is a limit to the number of targets against which drug candidates can be screened. Thus, phase I/II trials may reveal unanticipated toxicities. Adverse effects that become dose-limiting are of particular concern in the treatment of cancer. Nevertheless, deciphering the mechanistic basis of an adverse effect is an opportunity to uncover important regulations not appreciated previously. HSP90 inhibitors represent a novel treatment modality aimed at tumours with cells relying on HSP90 activity. Hyponatremia is a surprising side effect for patients exposed to an HSP90 inhibitor. The prime, if not sole, plausible candidate for a mechanistic explanation is vasopressin or vasopressin-dependent signalling. Vasopressin by activating the renal V2-receptor controls the concentration of sodium in the extracellular space. Here we investigated the mechanistic basis of hyponatremia by exploring the possibility that HSP90 inhibition enhanced signalling via the V2-receptor. Our experiments unequivocally demonstrated that HSP90 inhibitors increase the sensitivity of cells to vasopressin by raising V2-receptor levels based on the following observations. (1) Incubation of cells with an HSP90 inhibitor resulted in upregulation of the V2-receptor. Antibody-labelling of the receptor and binding of [^3^H]AVP indicated consistently that the increment was about twofold. The effect was seen with three inhibitor compounds, which differed in structure and affinity. (2) The upregulated receptors were fully functional; the increased surface density translated into commensurately enhanced receptor-mediated cAMP formation. (3) Enhanced V2-receptor signalling was independent of the cell type employed and was seen regardless of whether receptor expression was driven form the endogenous promoter in HELA cells or from the viral promoter provided by the plasmid used for heterologous expression of the receptor. (4) Depletion of HSP90 by siRNA-mediated downregulation recapitulated the effect of HSP90 inhibitors on V2-receptor levels.

The increased surface-receptor density and enhanced receptor-mediated cAMP formation is the plausible mechanism underlying the development of hyponatremia. Previously, Caltabiano and Kinter reported an experiment in normal rats chronically infused with a V2-receptor antagonist; the treatment led to upregulation of renal V2-receptors with significantly lower urine volume and increased urine osmolality [20]. Similarly in patients with nephrogenic diabetes insipidus (NDI), V2-receptor upregulation led to less voluminous, more concentrated urine [21]. Both of these observations underpin the consequence of an increased V2-receptor density for water re-absorption. Relative to the situation in patients administered an HSP90 inhibitor, the finding from the animal experiment probably is more relevant than are the results from patients treated for NDI. In the latter case receptor levels are below a certain threshold limit where modest upregulation suffices to generate a V2-receptor signal quantum and restore vasopressin responsiveness [21].

Based on the pertinent evidence it is safe to say that V2-receptor stimulated cAMP formation represents the appropriate surrogate parameter to predict increased renal water re-absorption. Cyclic AMP formation is the first signalling step in recruiting aquaporin 2 to the apical membrane patch of renal duct epithelia (reviewed in [22]). Cyclic AMP activates protein kinase A to phosphorylate aquaporin 2 (on serine residue 256), which initiates appropriate positioning of the water channel. Since cAMP is the relevant second messenger, prostaglandin E2 stimulates antidiuresis via Gs-coupled EP4 receptors present on collecting duct epithelial cells. Prostaglandin E2 regulates cAMP formation and urine concentration but it does not supplant the dominant role of vasopressin [23]. Our finding in cell culture that HSP90 inhibition increased the number of functional V2-receptors supports a cause-and-effect relationship.

We rule out that V2-receptor upregulation by HSP90 inhibitors results from an off-target effect. First, depletion of cytosolic HSP90, of both isoforms α and β, reproduced the effect of the inhibitors. With partial HSP90 depletion, the inhibitor effect diminished proportionally. Secondly, the effect sizes were in the same range with each HSP90 inhibitor if estimated from the increment in receptor density or receptor-mediated cAMP formation. Third, the concentration-dependence of receptor upregulation reflected the inhibitory potencies of the three chemically unrelated HSP90 ligands. Our data indicated EC50 values in the nanomolar, the submicromolar and micromolar concentration range for luminespib, DMAG and radicicol, respectively. These estimates are in agreement with values published in the literature [19, 24]. For radicicol, the potency varies with the cell type used to gauge the effect of HSP90 inhibition and is deemed lower in HEK293 culture than in others [16, 25, 26]. The model cells we have used were either resistant (HELA) or moderately susceptible (HEK293) to the cytotoxic effect of HSP90 inhibition. In our hands, choriocarcinoma cells (JEG-3 cell line) were affected the most and significant fractions of the culture were lost following incubation with HSP90 inhibitor. Similar to the HEK293 culture, however, a cAMP assay suggested that the heterologous V2-receptor was more active in treated than in untreated JEG-3 cells (data not shown). Thus, receptor upregulation was unrelated to cytotoxicity in cells treated with an HSP90 inhibitor.

Cytosolic HSP90 acts as molecular chaperone and assists the folding of client proteins. Many protein kinases and transcription factors depend on an association with HSP90 for their activity and stability. HSP90 can also control the level of transmembrane proteins at the cell surface: this has been demonstrated for G-protein-coupled receptors, e.g. the α2C-adrenergic receptor [15] and the A2A adenosine receptor [16] and for transporters, e.g. the renal Na^+^/Cl^−^-symporter/SLC12A1 [27] and the serotonin transporter/SLC6A4 [28]. In those instances, where the mechanistic details were examined, HSP90 was found to act in a relay with other heat-shock proteins at the level of the endoplasmic reticulum [16, 27, 28].

Along their biosynthetic pathway, G-protein-coupled receptors (GPCRs) are subject to a stringent quality control [29]. Inhibition of HSP90 can relax quality control, which may translate into increased export of GPCRs from the endoplasmic reticulum and hence augment receptor levels at the cell surface [15, 16]. It is not clear, however, whether this is the case with the V2 vasopressin receptor. There is also precedent for an action of HSP90 on later steps in GPCR trafficking [30]. Regardless of the underlying mechanism, our observations provide evidence that receptor upregulation causes hypersensitivity to vasopressin. For water retention to occur, an additional stimulus must shortcut feedback regulation to prevent a decline of the hormone. Consequently, activation of the V2-receptors present at increased density will raise cAMP to a level incommensurate with the physiological requirement.

The vasopressin feed back loop likely accounts for the observation that many more patients treated with an HSP90 inhibitor in clinical trials were unaffected than affected by hyponatremia. In patients with no evidence of hyponatremia, secretion of vasopressin presumably adjusted to the excessive sensitivity of renal V2-receptor signalling. In affected patients, however, an additional trigger must uphold hormone secretion to cause water retention. For example, a fall in blood pressure may act as a trigger; low blood pressure can powerfully increase vasopressin secretion despite normal serum osmolality.

Table 1 also includes two trials where the hyponatremia incidence rate was exceptionally high amounting to about 50% of the number of patients enrolled [5, 12]. In these trials, the HSP90 inhibitor was applied in combination with another anti-tumour drug—either erlotinib or docetaxel. The drug combination apparently enhanced the chance of developing hyponatremia. This suggests some sort of synergism, as erlotinib and docetaxel have rarely been associated with hyponatremia outside situations with a known hyponatremia incidence risk, as incurred by patients with non-small cell lung cancer or patients treated with cisplatin (the erlotinib-combination trial in fact included patients with lung cancer, but hyponatremia occurred exclusively in patients administered the HSP90 inhibitor at high dose [12]). Why docetaxel has this ability, is unclear. For erlotinib, however, a recent report demonstrated that blocking the EGF-receptor promotes aquaporin 2-mediated water re-absorption in a manner independent of V2-receptor signalling [31]. This finding invites the interpretation that—by upregulating the V2-receptor and initiating aquaporin 2 translocation—the EGF-receptor inhibitor erlotinib in combination with an HSP90 inhibitor maximized susceptibility to vasopressin. In this situation, vasopressin should be effective even at rather low levels.

If hypersensitivity to vasopressin is considered causal, vasopressin receptor antagonists appear a logical treatment option. Current guidelines however advise against the use of V2-receptor antagonists in patients with acute hyponatremia presumably including those treated for cancer [32]. Another reason for caution is that experiments on select tumour cell lines have suggested an inhibitory effect of the hormone. Recently for example, Pifano et al. [33] reported on an anti-proliferative effect mediated by the V2-receptor in a tumour cell xenograft model. Such observations would argue against the use of a V2-receptor antagonist in cancer patients; yet there is no evidence in the literature that treatment with an antagonist promotes growth or spread of cancer.

As per the original concept, HSP90 makes for an exquisite target in tumour cells addicted to HSP90, which implies that an inhibitor should effectively suppress tumour growth at submaximal concentrations. A conclusion from our data—in conjunction with the results from clinical trials—is that maximal concentrations give rise to unexpected adverse effects because they may interfere with many a function cytosolic HSP90 has in cell biology.
